# The Two Sides of Indoleamine 2,3-Dioxygenase 2 (IDO2)

**DOI:** 10.3390/cells13221894

**Published:** 2024-11-16

**Authors:** Chiara Suvieri, Maria Laura Belladonna, Claudia Volpi

**Affiliations:** Section of Pharmacology, Department of Medicine and Surgery, University of Perugia, 06129 Perugia, Italy; chiara.suvieri@unipg.it (C.S.); marialaura.belladonna@unipg.it (M.L.B.)

**Keywords:** indoleamine 2,3-dioxygenase 2 (IDO2), cancer, signaling function, tryptophan metabolism, indoleamine 2,3-dioxygenase 1 (IDO1), apo-enzyme

## Abstract

Indoleamine 2,3-dioxygenase 1 (*IDO1*) and *IDO2* originated from gene duplication before vertebrate divergence. While IDO1 has a well-defined role in immune regulation, the biological role of IDO2 remains unclear. Discovered in 2007, *IDO2* is located near the *IDO1* gene. Because of their high sequence similarity, IDO2 was initially thought to be a tryptophan (Trp)-degrading enzyme like IDO1. Differently from what expected, IDO2 displays extremely low catalytic activity toward Trp. Nevertheless, many studies, often contradictory, have tried to demonstrate that IDO2 modulates immune responses by catabolizing Trp into kynurenine, an unconvincing hypothesis linked to an incomplete understanding of IDO2’s activity. In this study, we review IDO2’s functional role beyond Trp metabolism. IDO2’s evolutionary persistence across species, despite being almost inactive as an enzyme, suggests it has some relevant biological importance. *IDO2* expression in human normal cells is poor, but significant in various cancers, with two prevalent SNPs. Overall, the comparison of IDO2 to IDO1 as a Trp-degrading enzyme may have led to misunderstandings about IDO2’s true physiological and pathological roles. New insights suggest that IDO2 might function more as a signaling molecule, particularly in cancer contexts, and further studies could reveal its potential as a target for cancer therapy.

## 1. Introduction

Gene duplication is a key process for acquiring new genes with new functions. This cell-life phenomenon is very common in all three domains, to the point that duplication is estimated to be as frequent as the rates of single nucleotide polymorphisms [1]. The existence of two copies for the same gene allows one copy to accumulate mutations while the other retains its original function. In many cases, the mutation-containing gene becomes a pseudogene; however, it can also evolve as a new functional gene [2,3].

The proteins indoleamine 2,3-dioxygenase 1 and 2 (IDO1 and IDO2, respectively), were identified as products arising from a gene duplication that occurred before the divergence of vertebrates [4]. Although IDO1 was discovered first, the indoleamine 2,3-dioxygenase found in lower vertebrates seems to be much more similar to IDO2 than to IDO1 [4]; thus *IDO2* is probably the original gene and *IDO1* its evolutionary product that has acquired new properties and functions, which were absent or only present as a draft in *IDO2*. Despite its ancestral origins, the biological functions of IDO2 are still undefined and under investigation. On the contrary, very soon after its discovery, IDO1 was assigned a pivotal role in the mechanisms controlling the immune response, primarily because of its ability to degrade tryptophan (Trp) and produce metabolites that contribute to immune tolerance [5]. This mechanism is particularly relevant in cancer, since tumors exploit IDO1’s activity to escape immune surveillance [6]. Based on this knowledge, IDO1 has been considered a therapeutic target, and several inhibitors have been designed aiming to block its catalytic activity, restore immune system function, and improve cancer immunotherapy outcomes [7,8]. In the wake of success in achieving IDO1 catalytic inhibition, attention has extended toward IDO2, considered an IDO1-related enzyme. Although IDO2’s role in immune regulation is less understood, efforts are underway to develop inhibitors targeting its enzymatic activity with the aim of broadening the scope of immunotherapeutic strategies [9]. Inhibition of IDO2 catalytic activity is being explored as a potential complement to therapies targeting IDO1 or using anti-PD-1 or anti-CTLA-4 antibodies to enhance antitumor response. While IDO1 inhibitors, although without concrete results so far [10], have hinted at some promise, the development of IDO2 inhibitors has not yet yielded definitive results. Research studies suggest that blocking IDO2 could improve immune response by reducing tumor-induced immune suppression, however, clinical trials focused on IDO2 are still in the early stages, and their efficacy remains under investigation [11,12].

In this review, we summarize the current knowledge on human IDO2 (hIDO2) and describe two distinct interpretations of its biological activity, the first considering it as one of the three enzymes metabolizing Trp and a more recent perspective suggesting a more complex biological role. The literature regarding the characteristics and biological functions of IDO2 in mice is extensive, but describe conflicting and often indirect results, partly due to the long-standing lack of optimal reagents for the detection of the murine IDO2 (mIDO2) protein [13]. For this reason, in the present review, we focus on the information reported in scientific literature related to IDO2 in humans.

## 2. IDO2, an Enzyme in the IDO1’s Shadow

In 2007, several groups independently described the discovery of a gene [14,15,16], located in mice and humans on chromosome 8p12, just downstream the already well-known *IDO1* gene. The discovery of the *IDO2* gene by Ball and colleagues was driven by the screening of cDNA libraries for the identification of *IDO1*-like sequences [14]. In the meantime, Metz et al. cloned *IDO2* starting from partial *IDO1* structural homologies found downstream the *IDO1* gene [16]. In the same year, a third group of researchers, based on a phylogenetic analysis, described *IDO2* as an *IDO1* paralog and hypothesized that IDO2 and other low-activity IDO paralogs found in non-mammalian organisms could be considered as proto-IDO enzymes [15]. Regardless of the approach that led to the discovery of IDO2, the high amino acid sequence homology with hIDO1 (43% identical, 63% similar) pushed researchers to use the latter as the main parameter to investigate the physiological role of IDO2.

Since the beginning, the “personality” of IDO2 was outlined as overlapping with that of the better-known protein IDO1, as demonstrated by the name IDO2. In fact, IDO2 has been defined as “dioxygenase”, although it has negligible enzymatic activity [14]. However, before addressing the inconsistency between the name and the functional activity of IDO2, it is important to provide a brief introduction to IDO1 to highlight how, up to now, knowledge about IDO1 has influenced the understanding of IDO2 through analogy, leading to a misinterpretation of IDO2’s functions.

IDO1, in mice and humans, is a heme-containing protein, whose best-known activity is the enzymatic degradation of Trp. Besides its catalytic function, a signaling function has also been recently described for IDO1 and is currently being studied for better characterization [17]. IDO1 is endogenously expressed in immune and non-immune cells and tissues, as well as in neoplastic cells and in the tumor microenvironment [18,19], in a dynamic balance between a holo- and an apo-conformation. On the one hand, holo-IDO1 (namely, the protein containing the heme cofactor), has a metabolic function relying on its catalytic activity. Holo-IDO1, indeed, catalyzes the degradation of the amino acid Trp, leading to the production of N-formyl kynurenine. Both depletion of the essential amino acid Trp and production of the first of several bioactive compounds through the so-called “kynurenine (Kyn) pathway” are mechanisms of tolerance induction extensively reviewed elsewhere [20]. On the other hand, apo-IDO1 (namely, the protein without the heme cofactor) has a transducer activity mediated by its interaction with SH2-containing proteins, as the Src homology 2 domain phosphatases (SHPs), the phosphoinositide 3-kinase (PI3K), and the suppressor of cytokine signaling 3 (SOCS3) [21,22,23]. Therefore, IDO1 is described as a “moonlighting protein”, capable of multiple biological functions depending on the intracellular heme availability and factors stabilizing the protein conformation. The catalytic and signaling functions of IDO1 appear to be confined to mutually exclusive conformations of the protein, compatible with the holo- and apo-IDO1 forms [24]. Notably, the description of IDO1 as a moonlighting protein is very innovative, since until recently, the immunoregulatory role of IDO1 was traced back only to its activity as an enzyme capable of degrading Trp [21]. Despite such observations, the signaling function of IDO1 remains something that scientists have not yet fully deciphered.

Based on these premises, when the gene coding for *IDO2* was discovered in 2007, the high amino acid sequence homology with IDO1 appeared to be a convincing characteristic to hypothesize that IDO2 was a Trp-degrading enzyme like IDO1 [14,15,16]. For this reason, IDO2 was immediately defined as “the third Trp-metabolizing enzyme”, along with IDO1 and tryptophan 2,3-dioxygenase (TDO) [25]. Although this definition is still used, some important differences between this protein and IDO1 do exist, as highlighted by the literature describing the discovery of IDO2. An analysis of its Trp degradation rate demonstrated that hIDO2 exhibits highly limited Trp-degrading enzymatic activity when compared to its close paralog, IDO1 [16,26,27,28]. The reported *km* value for hIDO2 with Trp as a substrate is generally quite high (approximatively 6.8–9.4 mM), 100-fold higher than the physiological Trp concentrations, depending on the experimental conditions and specific studies [14,16,27,28,29,30], and reflects IDO2’s low affinity for this substrate. Despite IDO2’s negligible activity as an enzyme, it has been supposed that D-1-methyl-tryptophan could be a selective inhibitor of its catalytic function [16], a hypothesis that nowadays seems to be definitively refuted [27,28,31,32]. The absence of a significant Trp degrading activity operated by IDO2 prompted researchers to postulate alternative hypotheses justifying, in IDO2, the coexistence of amino acid sequence similarity and different enzymatic ability with respect to IDO1. Thus, the limited catalytic activity of IDO2 has been ascribed to several factors related to its structural and functional properties. First, IDO2 could have a reduced affinity for Trp compared to its paralog IDO1 because of sequence variations in its active site possibly affecting the substrate and/or cofactor binding. As suggested by Yuasa et al. [4], the presence of a widespread threonine (Thr) residue in a crucial position for heme binding (in the hIDO2, distal-Thr171) compromises the catalytic activity and identifies IDO2 as a low efficiency Trp-degrading enzyme completely conserved across vertebrates. Conversely, in hIDO1, there is a serine (Ser) residue in the crucial position for heme binding. The substitution of this distal-Ser167 with a Thr dramatically increases the *Km* of Trp metabolism and decreases the catalytic efficiency [4]. Intriguingly, this hypothesis is supported by the unusually low *Km* value of lizard IDO2 (about 80 μM), a unique IDO2 that, similarly to IDO1, has a distal-Ser (Ser171) instead of a distal-Thr [4]. Similarly, Austin et al. [33] proposed that, despite the high degree of conserved amino acid in hIDO1 and mIDO2 (and hIDO2, our note) heme-binding sites, substrate access to heme appears to be decreased in mIDO2 compared to hIDO1 due to steric hindrance by bulky amino acids at the “pore” entrance. Because of IDO2’s low ability to bind the heme cofactor, its three-dimensional structure could be compared to an enzyme “frozen” in its apo-form. In this analogy, when IDO1 acquires its “apo” conformation, it is incapable of producing Kyn from Trp but is active as a transducer [17]. Moreover, three amino acids (Phe226, Phe227, and Arg231, in the human protein) were deemed critical to maintaining dioxygenase activity in IDO1 [34]. While two of the three amino acids are conserved in hIDO2, Phe227 has undergone a substitution to Tyr231. Although a Phe-Tyr mutation is considered conservative in terms of amino acid structure and degree of nucleotide change, this mutation might make the hIDO2 heme-binding pocket less prone to accommodate heme and more susceptible to phosphorylation or sulfation [33]. This observation might be of pivotal importance to shed new light on the biological role of IDO2, as described in the following paragraphs.

The abovementioned structural differences between IDO1 and IDO2 might also suggest that IDO2 could have evolved to interact with alternative substrates [28], although this has not yet been clearly identified. Finally, the low catalytic efficiency of IDO2 toward Trp could depend on the presence or absence of specific cofactors or environmental conditions, such as particular pH levels or redox states [27,33]. However, the hypotheses on how IDO2’s structure and experimental settings could impact its catalytic efficiency, so far, have not yet been proven.

These combined factors contribute to IDO2’s overall reduced capability in Trp degradation and suggest that its primary function might lie not in the classical Kyn pathway, but elsewhere, potentially involving other physiological processes or signaling pathways. In our perspective, it is crucial to emphasize that, although the role of IDO2 remains somewhat unclear, it is a matter of fact that this protein, despite its limited ability to degrade Trp, has evolved across different kingdoms with remarkable constancy [35]. This persistence suggests that IDO2 might have conserved essential functions or characteristics, having contributed to its evolutionary stability beyond its putative enzymatic role in Trp metabolism. Conversely, one might speculate that IDO1 developed its very efficient Trp degradation ability only as a duplication product of the ancestral IDO gene.

## 3. Enigmatic IDO2 and Where to Find It

The expression of *IDO2* in human cells is quite limited and less well-characterized than *IDO1*’s expression. Since 2007, the existence of two alternatively spliced transcripts for IDO2 had been described, coding one for a 407- [14], the other for a 420-amino acid (aa) protein [16]. The latter includes a 13-aa extra sequence [16] and is reported to be an IDO2 isoform lacking enzymatic activity in in vitro assays [29,36]. Nevertheless, starting from February 2024, major databases have retained only the coding sequence for the 407-aa form (i.e., NCBI reference sequence NM_194294.5), as the transcript for the protein containing thirteen extra amino acids was removed.

In normal tissues, *IDO2* mRNA expression is typically very low and restricted, with physiological expression primarily observed in the liver and placenta [37]. Moreover, during pregnancy, IDO2 is continuously expressed in syncytiotrophoblasts from the first trimester until term, with some additional expression in extravillous trophoblasts [38,39].

However, in cancerous tissues, *IDO2* expression becomes more pervasive. The overview derived from the TCGA dataset shows a wide range of *IDO2* expression among different cancer types [37], including non-small cell lung cancer (NSCLC) [40], glioma [41], colon, gastric, and renal tumors [9,32], and medullary thyroid carcinoma [42], with the highest median expression in diffuse large B-cell lymphoma and the lowest median expression in acute myeloid leukemia [43]. This observation has also been recently demonstrated in a large cohort of patients affected by various tumors [44]. In some tumors, IDO2 expression has also been observed in non-tumor tissues adjacent to the neoplastic masses [45]. Similarly, several human tumor and non-tumor cell lines express *IDO2* mRNA, such as the lung cancer cell lines A549 [46], H1650, H2228, H1975, and CALU-3 (our unpublished observations), but also MCF-7 [47], HEK293, HepG2, and CACO2 cell lines derived from tissues other than the lung’s (our unpublished observations and [48]). The expression of IDO2 in trophoblasts could suggest a potential pro-tumorigenic role like that observed in tumor environments, and maybe support the “trophoblast model of cancer” hypothesis [49]. In this model, the invasive and immune-modulating properties of trophoblasts, essential for pregnancy, are paralleled by tumor cells’ ability to evade immune detection and promote tissue invasion [49]. IDO2 could therefore represent a key mechanism that both trophoblasts and cancer cells exploit to escape regulatory control, highlighting IDO2 as a potential target for cancer therapies aimed at disrupting these shared pathways.

Besides the mRNA expression, several groups have recently demonstrated the presence of the IDO2 protein in surgical specimens obtained from patients affected by different types of cancer. Immunohistochemistry analyses demonstrated a significant presence of IDO2 in NSCLC [40], medullary thyroid carcinoma [42], B and T cell lymphomas [50], ovarian cell carcinoma [51], and glioblastoma [52]. Despite the extensive literature describing the presence of IDO2 in tumor cells, so far there is no certain information about its function in cancer onset/progression in humans. Although the biological role of IDO2 in mice is still very uncertain and debated [53,54,55], some clues regarding the hIDO2 function in cancer could come from the literature on mIDO2 in tumor models (Table 1). Indeed, it was demonstrated that IDO2 affects the proliferation, migration, and survival of murine tumor cells. The deletion of *Ido2* reduced the tumor volume in a mouse model of Lewis lung carcinoma [56]. Similarly, *Ido2* gene silencing slowed B16-BL6 cell proliferation, significantly inhibited tumor cell migration and tumor growth, and affected cell cycle phases both increasing and decreasing cell accumulation in G1 and G2/M, respectively [57]. These pieces of evidence suggest that IDO2 plays a role in cancer biology, particularly in cell cycle regulation, tumor progression, and cell proliferation. Its specific role and mechanisms are still being elucidated, but the IDO2 involvement in various neoplastic diseases makes this protein a promising target for future cancer therapies, and a potential biomarker and prognostic factor as well.

The expression of IDO2 in cancer cells could be of pivotal importance in driving the immune response against cancer in the context of an intriguing mechanism described by Sørensen et al. [66], who demonstrated the existence of spontaneous cytotoxic T lymphocyte reactivity against IDO2 in the peripheral blood of cancer patients. Within the IDO2 protein, they identified HLA-A2 peptides targeted by spontaneous T-cell reactivity in patients suffering from unrelated tumor types. Surprisingly, healthy individuals also hosted spontaneous immunity against IDO2 [66]. Although the cytolytic effect of IDO2-specific class I-restricted lymphocytes in peripheral blood of healthy subjects against IDO2-expressing cells has still to be proven in vivo, Sørensen et al. hypothesize that the sizable reactivity to IDO2-derived antigens in normal individuals contributes to immune surveillance against cancer [66]. It remains unclear how anti-IDO2 reactivity observed in healthy individuals could be justified. While IDO2 involvement in immune regulation has been described in mice [60], IDO2 expression is absent in the majority of normal cells in humans [37]. This discrepancy raises questions about the mechanisms driving the immune system to target IDO2 under non-pathological conditions. Further studies are needed to elucidate whether this reactivity is linked to specific contexts, such as latent autoimmune responses, or if other unknown factors are actively involved. Beyond these arguments, the limited expression of IDO2 in normal tissues reinforces its potential as a selective target for therapeutic intervention against cancer [46].

Despite the prominent role of the paralog IDO1 in immune cells (besides cancer and tumor microenvironment cells) [20], little is known about the expression and the role of IDO2 in the human immune system. Silencing *Ido2* in murine dendritic cells inhibits the tumor growth in vivo, promotes the proliferation of T lymphocytes, and reduces the formation of regulatory T cells in vitro [67]. In human myeloid and plasmacytoid dendritic cells, the expression of *IDO2* has been documented since its discovery [31,68], but it has also been demonstrated that it lacks any enzymatic activity [31], an observation leaving unclear IDO2’s functional role in these cells, both in the physiologic and in the neoplastic context. Overall, despite several attempts to understand it, the biological function of IDO2 is still completely unknown. Using a HEK293 cell line overexpressing both *IDO1* and *IDO2*, Lee et al. [69] investigated the influence of IDO2 on IDO1 catalytic activity to assess the interplay between these two proteins and hypothesized that IDO2 could play a role as a negative regulator of IDO1 by competing with it for heme-binding. However, it is not yet known whether this mechanism is exploited in any physiological or pathological context.

In contrast to IDO1, whose primary inducers are known [70], little—and occasionally contradictory—information is available about the stimuli that can regulate IDO2 gene expression. On one hand, IFN-γ is the most prominent inducer of the *IDO1* gene [71]; on the other hand, although several studies demonstrate that IFN-γ could induce the expression of *IDO2* in human mesenchymal stem cells and some cancer cells [72,73], in human dendritic cells, the cytokine is ineffective [74]. Moreover, the upregulation of *IDO2* was found to be induced by IL-10, prostaglandin E2, and lipopolysaccharide [68,75,76]. Interestingly, the aryl hydrocarbon receptor (AhR) can also induce the expression of *IDO2*, suggesting that there is an AhR responsive element in the promoter of the *IDO2* gene [74,77].

Aside from their mRNA expression, which is controlled by distinct inducers, IDO1 and IDO2 proteins also have different levels of stability. IDO1 is continuously transcribed, translated, and degraded, in contrast to IDO2, whose protein, once synthesized, appears to be stable [68]. Indeed, IDO1 contains one tyrosine (Tyr) residue within each of the two canonical immunoreceptor Tyr-based inhibitory motif (ITIM) sequences that direct protein turnover through SOCS3-mediated ubiquitination and proteasomal degradation [23]. Like IDO1, hIDO2 possesses two ITIM domains. However, while the so-called ITIM2 shows a high homology sequence and a similar position in both the IDO1 and IDO2 proteins (VYEGF in the hIDO1 and MYEGV in the hIDO2), the putative IDO2’s ITIM1 motif (IFYAGI) differs from IDO1’s (VPYCQL) [46]. Moreover, although the ITIM2 domain is conserved in IDO1 and IDO2, only IDO1 is degraded by the proteasome, with its ITIM2 being specifically targeted by SOCS3 [23]. On the contrary, IDO2 appears to be neither able to bind SOCS3, nor to be degraded via the proteasome [68]. As discussed below, SOCS3 is only one of IDO1’s molecular partners, not interacting with IDO2 at all, a condition that does not help our understanding by analogy of the biology and functional role of IDO2. The main differences between hIDO1 and hIDO2 are outlined in Table 2.

## 4. One, None, and Hundred Thousand IDO2

A peculiar feature of the *IDO2* gene is the high prevalence of the two SNPs rs10109853 and rs4503083, identified by Metz et al. during the first characterization of IDO2 cDNA [16]. In this study, the authors described hIDO2 as a 420-aa protein, with the first SNP corresponding to a C-T substitution converting the Arg248 into Trp, and the second SNP consisting of a T-A nonsense mutation changing the Tyr359 codon into a premature STOP. Nowadays, after the removal of the 420-aa variant from databases, hIDO2 is commonly considered a 407-aa protein, lacking the first 13 aa, which are instead included in the sequence [16]. Nevertheless, the initial denomination of the two SNPs, namely R248W and Y359X, has been maintained instead of being changed into the more correct R235W and Y346X, as in Witkievicz et al. [73]. Aware of the mistake, in this review we use the original denomination.

Both SNPs have been described as “inactivating” the enzyme, since they completely abrogate the already negligible enzymatic activity of IDO2 [16]. From this perspective, the presence of one or both SNPs in various pathological contexts has always been assimilated to the presence of a “non-functioning” IDO2. In our view, since the actual mechanism of action of IDO2 is still unknown, one could speculate that the two SNPs would affect disease settings not involving the Trp catalytic function, so that, when the physiological role of IDO2 is deciphered, it will be necessary to reinterpret many literature data. Whatever their meaning, large scale sequencing analysis has revealed that these two nonfunctional alleles of *IDO2* are frequently distributed in human populations of Asian, European, and African descent; thus, the presence of R248W and Y359X has been evaluated as a possible prognostic/diagnostic factor in many tumor types, as well as in various other pathologies.

As for other neoplastic diseases, IDO2 is frequently upregulated in human pancreatic ductal adenocarcinoma (PDAC) [65,73]. The analysis of the two *IDO2* SNPs’ prevalence, combined with the treatment outcomes, indicated that in PDAC patients having received adjuvant radiotherapy, the “IDO2-inactive status” is significantly associated with improved disease-free survival [65]. Moreover, female patients with PDAC rarely harbor the IDO2-deficient status. In NSCLC patients, it has been revealed a highly significant incidence of the R248W genotype compared to the control group [54], as well as strong evidence of a significant correlation between *IDO2* expression and poor NSCLC prognosis [40]. Among the analyzed lung cancer histotypes, adenocarcinomas showed the highest IDO2 expression associated with high intratumoral/mixed tumor-infiltrating lymphocyte localization. In the same study, 83% of tumors showed a membrane reinforcement staining of IDO2 that, in 51% of cases, localized at the basolateral side of the cell membrane between tumor and stromal tissue. The genetic features of cancer patients’ IDO2 might also drive their immune response. As demonstrated by the association analysis between spontaneous IDO2-specific T-cell responses and the *IDO2* genotype of melanoma, renal cell carcinoma, and breast cancer patients, the induction of IDO2-specific T cells in peripheral blood is restricted to individuals that are not homozygous for the stop codon [79]. Furthermore, stronger T-cell responses occurred in patients with the wild type Tyr359 homozygous when compared with the heterozygous genotype. Interestingly, a higher number of immune responses against IDO2 also occurred in patients homozygous for the Trp248, compared with the Arg248 [79]. The meaning of these data might be revisited in the future, since, in the opinion of Køllgaard et al., spontaneous immune responses against IDO2 are associated with a reduced enzymatic activity of IDO2 itself [79], according to the theory that the R248W SNP would abrogate the catalytic function of IDO2 and “inactivate” the protein. Indeed, Køllgaard et al. proposed that the enzymatic activity of IDO2 could influence the systemic adaptive immune response and, therefore, IDO2 expression in the tumor target cells could suppress the cytotoxic T-cell responses and vanish the immune reactivity against the potential cancer antigen IDO2 [79]. The allelic status of *IDO2* has also been evaluated in patients with glioma treated with chloroquine (CQ) [80]. Prompted by their own unpublished observation that CQ is a potent and selective inhibitor of IDO2, and following the hypothesis that *IDO2*-inactivating SNPs could blunt clinical responses to CQ therapy, in a pilot study, Eldredge et al. evaluated the response to the combined treatment of whole brain radiotherapy and CQ administration in patients with brain metastases, stratified by *IDO2* genotype [80]. Although a trend toward improved overall survival was observed, there was no appreciable difference between patients with wild-type *IDO2* compared with enzymatically ablative SNPs [80]. Again, the awareness that the enzymatic activity of IDO2 is almost certainly not decisive for its physiological or pathological function could prompt a review of these data in a new light.

Many pieces of evidence show for IDO2 a sometimes-conflicting role in mediating inflammatory/autoimmune responses, especially in murine models of psoriasis, arthritis, and contact hypersensitivity [53,54]. However, in humans, an IDO2 functional role in mediating inflammation and driving B cell production of autoantibodies has never been proven, and has instead only been demonstrated in mice. In patients suffering with relapsing-remitting multiple sclerosis (MS), no difference in the expression of *IDO2* was recorded in comparison to healthy controls [81]; moreover, in Italian MS patients, it has been demonstrated that *IDO2* rs10109853 and rs4503083 polymorphisms are not associated with MS risk, age at onset, or disease progression [82]. Lee et al. investigated the correlation between *IDO2* genotype and the phenotype of Crohn’s disease patients, considering five *IDO2* SNPs (namely, rs4503083, rs4736794, rs10109853, rs35212142, and rs35446289) [83]. However, although *IDO2* minor allele variants are common and one of them, rs45003083, is associated with reduced risk of Crohn’s disease, none of the *IDO2* SNPs is associated with a particular Crohn’s disease clinical phenotype [83]. The expression of *IDO2* seems to be very high in lung tissues from patients who died with COVID-19 [84]. Interestingly, macrophages, dendritic cells, and neutrophils also showed IDO2 staining, but lymphocytes and mast cells did not [84]; moreover, since immunohistochemistry staining showed colocalization of IDO2 and several Trp metabolites, as well as AhR, Guo et al. hypothesize that systemic and early *IDO2* expression and activity via AhR would result in fatal cellular stress in severe COVID-19 [84]. However, this hypothesis is not sustained by a functional demonstration of IDO2’s capability to activate the Kyn pathway; therefore, convincing evidence of any IDO2 involvement in the complications due to SARS-CoV-2 infections is still missing. One possible explanation for the seemingly disparate outcomes observed in many settings of inflammation and autoimmune diseases is that IDO2 activity could be exclusively linked to the physiopathologic context and cellular milieu. This theory is supported by a study that found distinct patterns of *IDO2* SNPs in two distinct cohorts of individuals with aspergillosis. More precisely, the R248W and Y359X genotypes did not correlate with a higher risk of aspergillosis in patients with cystic fibrosis, but these same SNPs were necessary for the best antifungal activity in patients undergoing hematopoietic stem cell transplantation [85]. Finally, it has been demonstrated that the distribution of the two common *IDO2* SNPs in the Chinese population is overlapping with the already known distribution, while there is no significant difference in the distribution of genotypes between healthy subjects and patients with tuberculosis [86].

## 5. Viewing IDO2 in a New Light to Discover Its Biological Function

Overall, the above reported observations suggest that comparing IDO2 to IDO1 as a Trp-degrading enzyme has led to the misinterpretation of its physiological and pathological roles. IDO1 and IDO2, despite sharing structural similarities, might exhibit distinct regulatory mechanisms and biological effects, still to be completely clarified. The prevalent focus on IDO1 has overshadowed the functional contributions unique to IDO2, which might operate under different contexts or conditions. The result has been an incomplete understanding of IDO2’s biological role, leading to potentially flawed conclusions about its involvement in various diseases and physiological processes. The difficulty in deciphering the function of IDO2 also derives from the lack of an hIDO2 crystal and, consequently, of structural information.

**Figure 1:**
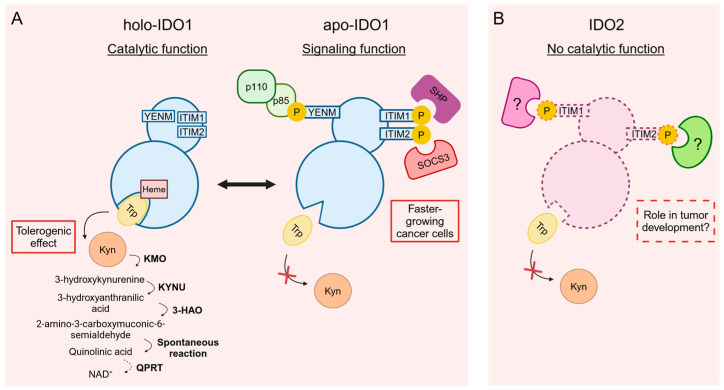
Comparison of IDO1 and putative IDO2 functions in neoplastic cells. (**A**) The two conformations dynamically acquired by IDO1. The IDO1 conformation containing the heme cofactor (holo-IDO1) is associated with significant activity of tryptophan (Trp) catabolism. In a tumor context, holo-IDO1 can trigger a tolerogenic effect depending on both Trp starvation and production of kynurenine (Kyn), besides the generation of other immunosuppressive downstream catabolites along the Kyn pathway. In contrast, the IDO1 conformation not binding the heme cofactor and catalytically inactive (apo-IDO1) undergoes tyrosine-phosphorylation of specific domains containing YENM and ITIM motifs, more accessible in apo-IDO1 compared with holo-IDO1. After phosphorylation, they act as docking sites for molecular partners such as the p85 catalytic subunit of PI3K, SHP phosphatases, and SOCS3, able to direct localization, function, or proteasomal degradation of IDO1. In cancer cells, the apo-IDO1 protein can promote the tumorigenic phenotype and increase cancer growth. (**B**) What is so far known and hypothesized about IDO2 conformation and function. Based on its negligible Trp-catalytic activity, IDO2 conformation might resemble the apo-IDO1 protein. The presence of ITIM domains, putatively protruding and tyrosine-phosphorylated as in the apo-IDO1 protein, could support the hypothesis of an IDO2 protein lacking the Trp-catalytic activity, but able to mediate a signaling function in tumor cells. IDO2’s interaction with possible molecular partners and its involvement in tumorigenic pathways is still to be elucidated, as shown in (**B**) by the presence of question marks. Figure created with BioRender.com. KMO: kynurenine 3-monooxygenase; KYNU: kynureninase; 3-HAO: 3-hydroxyamino oxidase; QPRT: quinolinate phosphoribosyl transferase. Solid lines: already described conformations and functions; dashed lines: hypothetic conformations and functions.

In our view, a more effective approach to understanding IDO2’s role could be, rather than solely focusing on its enzymatic activity, comparing IDO2 to the apo-form-folded IDO1, which is endowed with signaling functions (Figure 1). By examining IDO2 within the broader context of IDO1’s signaling pathways, we might gain deeper insights into its involvement in physiological and pathological mechanisms. Indeed, we have recently demonstrated that, in a lung adenocarcinoma cell line, IDO2 exhibits a peculiar membrane localization [46] and is basally phosphorylated in a still unknown Tyr residue [46]. Similarly, the subcellular localization of IDO1 as a signaling molecule is in the early endosomes’ membrane [70], where this protein is anchored by the binding to class I phosphoinositide 3-kinases [22]. Since ITIM motives are present in both IDO1 and IDO2, with one of them (namely, ITIM2) conserved in the two proteins, ITIM-binding molecular partners could potentially be another similarity. Instead, SHP-1 and SHP-2 phosphatases, interacting with IDO1’s ITIM1 to mediate immunoregulatory IDO1 signaling activity in DCs, do not interact with IDO2 [54]. Nevertheless, SOCS3, the main mediator of the proteasomal degradation of IDO1, binds IDO1’s ITIM2 [24], but is incapable of interacting with the corresponding and well conserved ITIM2 motive of IDO2. Moreover, the IDO1’s YENM motif—a consensus sequence for the binding of to the PI3K—is absent in IDO2 [22,54]. However, an element of similarity relies in the ability of Src kinase to phosphorylate both IDO1 and IDO2 [46,87], though it is still to investigate completely whether the Src-mediated phosphorylation of IDO2 is exploited in any physiological or pathological conditions. Interestingly, the SNP reported to irreparably affect the catalytic activity of the hIDO2, namely the substitution of the heme-binding Arg235 into Trp (known as R248W), could have a completely different biological outcome. Indeed, if we prioritize the signaling instead of the enzymatic role of IDO2, this SNP might conversely lead to a rise in biological activity, further diminishing IDO2’s ability to bind the heme group and tipping the scales in favor of its “apo-form”, putatively equipped with the signaling activity.

In conclusion, we propose an intriguing emerging perspective, suggesting that IDO2 might function as a signaling molecule, whose biological significance, still to be fully elucidated, should primarily be explored within the tumor context, where IDO2 prevalent expression has now been incontrovertibly demonstrated. Further research efforts and exploration of IDO2’s role could provide valuable insights into its potential as a target for therapeutic intervention in oncology.

## Figures and Tables

**Table 1:** Potential IDO2 role in different pathologies.

	Disease	Model	IDO2 Role	Reference
Autoimmunity/ inflammation	Arthritis	KRN TCR transgenic mice	Pro-inflammatory	[58,59,60]
	Psoriasis	Imiquimod-induced psoriasis-like dermatitis in mice	Anti-inflammatory	[61]
	Multiple sclerosis	MOG_35–55_-induced EAE	No effect	[62]
	Contact hypersensitivity	CHS (mouse)	Pro-inflammatory	[63]
	Endotoxic Shock	LPS (mouse)	Anti-inflammatory	[64]
Cancer	Melanoma	B16-BL6 mouse cell line, in vivo tumor model	Pro-tumoral	[57]
	Lewis lung carcinoma	LLC mouse model	Pro-tumoral	[56]
	PDAC	KRAS mouse model, IDO2 SNP analysis in patients	Pro-tumoral, IDO2 SNP associated to improved disease-free survival	[65]
	Breast cancer	Human MCF-7 cell line	Pro-tumoral	[47]
	NSCLC	Patients’ specimens	Poor prognosis	[40]
	Medullary thyroid carcinoma	Patients’ specimens	Poor prognosis	[42]
	Glioblastoma	Patients’ specimens	Poor prognosis	[52]

**Table 2:** Main differences between IDO1 and IDO2 in humans.

	hIDO1	hIDO2	Reference
Tissue mRNA expression in physiological conditions	Lung endothelial cells, secondary lymphoid organs, placenta	Liver, placenta	[37,78]
Regulatory stimuli	IFNγ, PGE2, LPS, IL-1β, IL-6, TGFβ, spermidine	IFNγ, IL-10, PGE2, LPS	[68,71,72,73,75,76]
L-Trp binding affinity (Km)	20.90 ± 3.91 μM	6809 ± 917 μM 9360 ± 810 μM	[28,29]
L-Trp catalytic constant (Kcat)	2.97 ± 0.20 s^−1^	0.103 ± 0.006 s^−1^	[28]
ITIM consensus motif	ITIM1	ITIM1	[21,24,46]
	VPY(111)CQL	IFY(231)AGI	
	ITIM2	ITIM2	
	LVY(249)EGF	LMY(253)EGV	
YENM (PI3K binding motif)	Y(145)ENM	Absent	[22,46]
Molecular binding partner	SHP1	Absent	[21,22,23,24]
	SHP2	Absent	
	SOCS3	Absent	
	PI3K	Absent	
Physiological role	Immune regulation via Trp-catalysis and signaling function	Not defined, low Trp enzymatic activity	[5,21,70]

## Data Availability

Not applicable.
